# Healthcare professionals’ experiences of providing care for patients with enterocutaneous fistula in hospital and in homecare: A qualitative study

**DOI:** 10.1371/journal.pone.0284782

**Published:** 2023-05-08

**Authors:** Karolina Härle, Sussanne Börjeson, Olof Hallböök, Pär Myrelid, Ingela Thylén

**Affiliations:** 1 Department of Surgery, Department of Biomedical and Clinical Sciences, Linköping University, Linköping, Sweden; 2 Department of Health, Medicine and Caring Sciences, Linköping University, Linköping, Sweden; 3 Department of Cardiology and Department of Health, Medicine and Caring Sciences, Linköping University, Linköping, Sweden; University of Stirling, UNITED KINGDOM

## Abstract

**Background:**

Enterocutaneous fistula is a rare but complex and serious condition that is both physically and psychologically demanding for the person affected. Infection, fistula dressing problems, electrolyte and fluid imbalance and malnutrition render the individual in need of long periods of in-hospital care and homecare. This place great demands on patients, families, and healthcare professionals. More research is needed to bridge the gap between hospital and home-based healthcare services.

**Aim:**

To explore healthcare professionals’ experiences of providing care for patients with enterocutaneous fistula in hospital and in homecare.

**Material and methods:**

A qualitative descriptive study design based on five focus group interviews involving 20 healthcare professionals. Data was analysed using content analysis.

**Results:**

Three categories with seven corresponding subcategories were developed; 1) Providing care for patients with enterocutaneous fistula in the hospital and at home was complex and time and resource consuming. Participants were struggling with practical issues and lacked disease-specific knowledge and skills; 2) Caring requires an integrated approach but it was challenging to work interprofessionally and cope with barriers in collaboration between providers; 3) Building long lasting relationships with patients and their families was fundamental to the caring process. Participants needed to hide their own feelings related to smell and appearance of the fistula as well as frustration when the fistula dressing did not hold without leaking. The healthcare professionals expressed that the involvement of the patient and their close family was important when proving care, likewise, to have a great understanding of the patients’ suffering.

**Discussion:**

The care of patients with enterocutaneous fistula is complex and means engagement for long periods, both in hospital and in home-based healthcare. Regular team meetings of the multidisciplinary team, careful planning before discharge and providing person-centred care can facilitate the care process.

## Introduction

An enterocutaneous fistula (ECF) is an abnormal connection between the intestine and skin. It is a rare but serious condition, difficult to treat and associated with high morbidity, high mortality and long periods of in-hospital care [1]. In more than 85% of cases, the aetiology of ECF is a complication of surgery. In the remaining 15% of cases, it occurs spontaneously due to an underlying disease, primarily Crohn’s disease [2]. Approximately 60% of all ECF close spontaneously within two months. In the remaining 40% reconstructive surgery is usually required [3]. However, reconstructive surgery cannot be undertaken until six to twelve months after the onset of the ECF due to the inflammatory response, nutritional status, and recovery of the abdominal cavity [3–5]. This places great demands not only on the patient and family, but also on healthcare professionals.

Living with ECF causes pouching problems, electrolyte and fluid imbalance, malnutrition and infections [5], which render the person dependent on healthcare [6,7]. Moreover, the condition is both physically and psychologically demanding for the individuals affected, which could lead to depressive symptoms and feelings of anxiety [6–8]. Thus, the patient needs support from both family and healthcare professionals in order to cope with the situation, as well as to promote motivation and self-care [6]. There is a great advantage if individuals with ECF can be cared for at home due to the risk of a poorer prognosis for those who need to be in hospital while waiting for surgical intervention [1]. The significant need for care means that careful planning before returning home is of great importance [5]. This complex situation means multidisciplinary teamwork is necessary [1,2,5,9]. However, a multidisciplinary team approach that provides care based on the needs and preferences of each patient irrespective of where the care is provided, can be challenging due to organisational barriers [7]. Nevertheless, there is a lack of studies reporting the experiences of providing care for patients with ECF and how to overlap those organisational challenges. More research is needed to bridge the gap between hospital and home-based healthcare services. Therefore, the purpose of this study was to explore healthcare professionals’ experiences of providing care for patients with ECF in hospital and in homecare.

## Methods

### Settings

Sweden is a country of 10 million inhabitants divided into 290 municipalities and 21 regions. The regions have ultimate medical responsibility for the provision of healthcare to citizens and the administration of care in the hospitals and primary healthcare centres. The municipalities have responsibility for the home-based healthcare; this involves non-physician medical interventions, rehabilitation, habilitation and nursing care undertaken by licensed healthcare professionals or other authorised staff, such as assistant nurses. Medical intervention is provided by physicians employed by the regions. Home-based healthcare is given to people who require healthcare for either a short or long period of illness but do not require hospitalisation [10]. This way of organising care is complex and provides challenges when planning, providing, and evaluating care for a patient in need of both hospital- and home-based healthcare. In addition, distance between the patient’s home and the home-based healthcare district can sometimes be more than 50 kilometres.

### Design

A qualitative descriptive study design with focus group interviews was undertaken [11]. A focus group means a group of people which are purposefully selected based on their background characteristics. Focus groups were used to obtain rich data because the individuals can elaborate on each other’s stories and help remember events and experiences that might not have been disclosed in an individual interview. A suitable analysis for focus group interviews is content analysis [11]. Therefore, data was analysed using qualitative content analysis as described by Elo and Kyngäs [12].

### Data collection

#### Study participants

The participants were selected through purposeful sampling [11,13] and included healthcare professionals from four county hospitals and three home healthcare districts. Nurses, enterostomal therapy nurses, physicians, and nurse assistants with experience of caring for patients with ECF were invited to participate. In total, five focus groups (n = 20) with three to five participants in each group were conducted. The participants’ characteristics are further described in Table 1.

**Table 1:** Participant characteristics.

Participant characteristics	N = 20
Gender, female	15
Age, years (range)	49 (23–79)
Healthcare profession	
Physician	3
Registered nurse	9
Assistant nurse	4
Enterostomal therapy nurse ^a^	4
Workplace	
Hospital	14
Home-based healthcare	6

^a^ Enterostomal therapy nurses are registered nurses who specialise in the management and care of patients with urinary and faecal diversions and/or incontinence.

#### The focus group interviews

Data was collected between June 2019 to April 2022. A semi-structured interview guide was used as a prompt to encourage discussion [11], based on a literature review and experiences from previous studies undertaken by the research group [6]. Before initiating the focus group discussion, participants answered question about demographics. Participants were informed about the aim of the study and rules during the interview, for example that participation in the focus group was voluntary, that it is okey to abstain from discussing specific topics if not being comfortable, not to interrupt each other and help to protect others’ privacy by not discussing details outside the group. The focus group discussion setup was guided by the principles outlined by Krueger and Casey [11] and the interview guide included opening, introductory, transition, key and ending questions (Table 2).

**Table 2:** The interview guide.

**Opening question**	Tell us your name, where you are working, your profession and in brief where you have met patients with enterocutaneous fistula.
**Introductory question**	Can you describe your experience of providing care for patients with enterocutaneous fistula?
**Transition and key questions**	What are the challenges in caring for these patients? What can improve care for patients with enterocutaneous fistula? What knowledge is necessary for healthcare professionals when providing care for patients with enterocutaneous fistula?
**Ending questions**	Of everything we have discussed today, what is the most important? Assistant moderator summary with following question: Is this summary consistent with your experience? Is there anything else that we have not discussed today that you want to talk about?

The opening question was intended to get people to start talking, followed by the introductory question that was about the experience of caring for patients with ECF. In transition and key questions, participants were asked to reflect on challenges in caring for these patients, what can improve care and what knowledge healthcare professionals need to care for patients with ECF. The ending question asked participants to reflect on the discussion and mention the most important topic from everything they had discussed. Interviews were moderated by an advanced nurse practitioner with research capacity (the first author) with help of an assistant moderator—also a nurse with experience of caring for patients with ECF. None of the moderators were working colleagues of the participants. During the interview the moderator asked follow-up questions such as: “Can you please tell me more about that?” or “What do you mean?” to encourage participants to elaborate on their stories and for clarification. At the end of the interview, the assistant moderator summarised the discussion and participants had the opportunity to accept, correct or expand to increase dependability. The assistant moderator took field notes during the interviews, for example about the topics discussed and level of participation. The field notes were not included in the analysis but were used for the summary at the end of each interview. Directly after the interview, the moderator and assistant moderator had a debrief on the discussion and the activity within the group. The moderator’s impression was that the activity in each group was lively with participants sharing their experiences with each other in a supportive way. The first interview was conducted as a pilot focus group, to test the interview guide and the roles of the moderators and as no significant changes were made, the pilot group was later included in the analysis. The interviews were undertaken in Swedish, audio-recorded and transcribed verbatim. Four of the interviews took place in a conference room in the respective hospitals and one of the interviews was performed digitally via Zoom. The interviews lasted between 47–78 minutes (mean 63 minutes).

### Analysis

Data was analysed by qualitative conventional content analysis as proposed by Elo and Kyngäs [12]. To facilitate immersion in the material and to make a sense of the whole, the analysis started with listening to the audio-recordings and several readings of the transcripts. During open coding, the data was carefully read line-by-line and all words, sentences or paragraphs that were related to the study aim were extracted and coded. In the categorisation phase, the codes were constantly compared to each other and sorted based on their belongings, which included both differences and similarities. The codes were then grouped together into subcategories, which were later sorted and merged into fewer, more overarching categories. In order to allocate the findings into the same category, the researcher used some degree of interpretation (i.e., went beyond the exact words and was open to the underlying meanings that were conveyed) when approaching the text. The first author undertook the initial coding and categorising and the last author validated and suggested changes. This was an iterative and non-linear process, moving back and forth between parts and the whole, until no new codes or categories merged [12]. The first author was careful to stay close to what the participants actually said, while also keeping in mind that an abstraction occurred through the different phases of the analysis. When all subcategories and categories were defined by the first and last author, these and the coding scheme were reviewed by a third author for agreement or discussion until a consensus was reached. Finally, each subcategory was strengthened by quotations for transparency [13]. The quotations in the result were translated verbatim from Swedish to English. Every quotation is stated along with the specific interview number and the participant’s profession with the following abbreviations: RN (registered nurse working in the hospital), HRN (registered nurse working in homecare), AN (assistant nurse working in the hospital), HAN (assistant nurse working in homecare), ET (enterostomal therapy nurse working in the hospital) and MD (physician working in the hospital). Table 3 is an illustrative example of the analytical process.

**Table 3:** Example of analytical process for data from focus groups.

Excerpt from unit of analysis	Open coding	Subcategory	Category
*“It can be terribly challenging and frustrating and… yes*, *sometimes you get completely desperate*. *You try and try different fistula dressings*, *sometimes a dressing takes up to two hours… and when it looks great you leave the patient and then half an hour later there is a leak again…”*	Challenging and frustration with the fistula dressing	Struggling with practical issues	Caring consumes time and resources

### Ethical considerations

Ethical approvals were obtained from the Regional Ethical Review Board (record no 2017/121-31). The study was undertaken in accordance with the principles of the Declaration of Helsinki [14]. Participants were informed that participation in the study was voluntary and that they could withdraw their consent to participation at any time without giving a reason. All material was treated confidentially. The audio-recordings and transcripts were coded by number. Before the interview, a verbal agreement was carried out that the discussion held within the group should remain confidential and not be shared outside the group.

## Results

**Fig 1:**
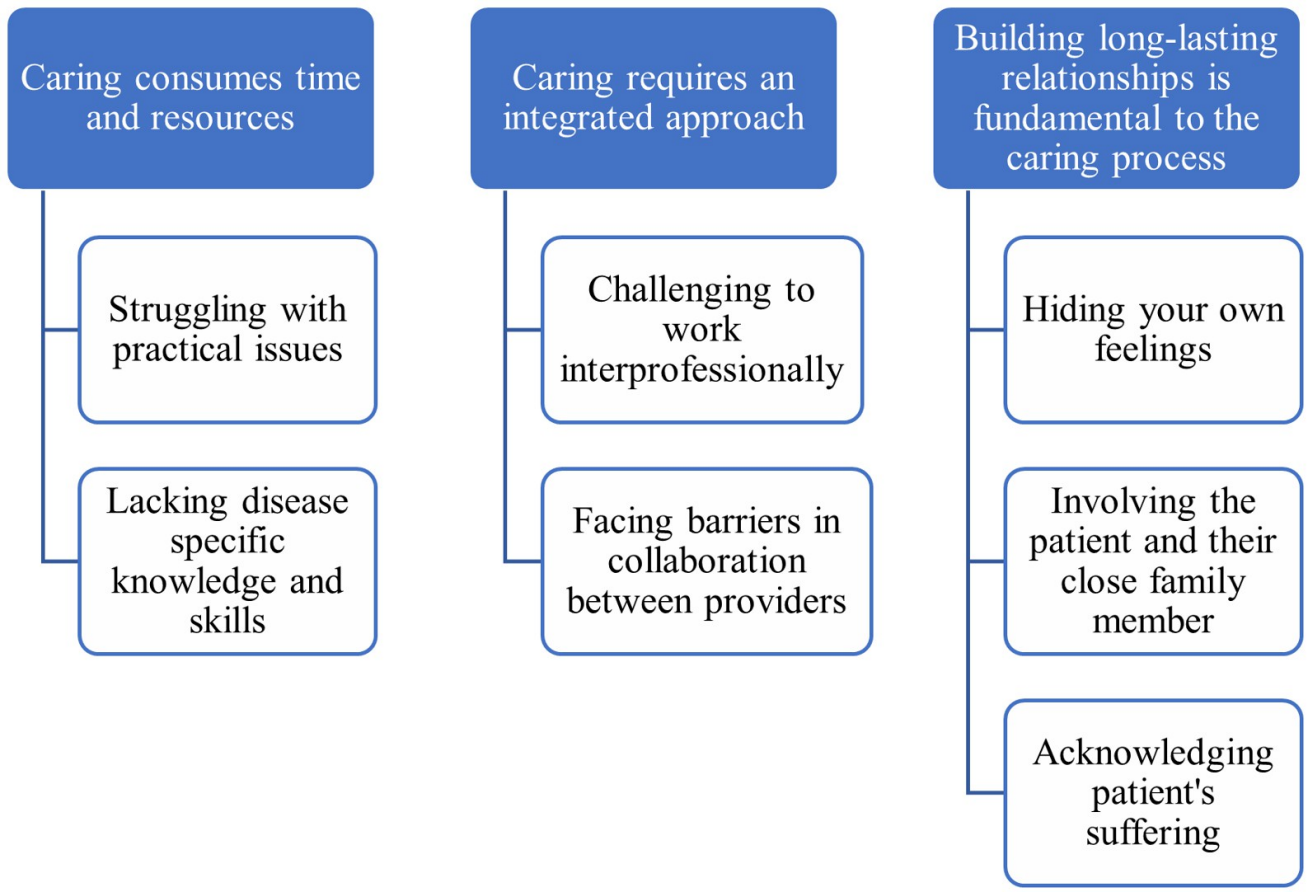
Categories and corresponding subcategories.

The findings show that caring for patients with ECF is complex and consumes time and resources, requires an integrated approach and that building long lasting relationships is fundamental to the caring process. In total, three categories with seven corresponding subcategories were developed to capture the healthcare professionals’ experiences of providing care for patients with ECF in the hospital and at home (Fig 1).

### Caring consumes time and resources

Patients living with an ECF were experienced as a complex group of patients with a high level of need for care and who often require prolonged hospital and home-based healthcare. The healthcare professionals struggled with a lot of practical issues that hindered the caring process. They also expressed that knowledge of ECF was necessary to facilitate care. Lacking disease-specific knowledge and skills was both time and resource consuming and for those who were knowledgeable, it was a challenge to be the sole expert.

#### Struggling with practical issues

Participants felt that patients with ECF were demanding on resources, with a lot of both medical and nursing problems. Patients were often in need of total parenteral nutrition and analgesics. Participants described often encountering challenges in finding a durable fistula appliance or a lack of appropriate appliance, but also individual factors because fistulae look different on different patients. Dressing a fistula was also very time consuming. Much of the frustration experienced by the healthcare professional in the care of these patients was related to the fistula appliance.

***ET***: *It is of course*, *it’s hugely resource intensive for nursing staff because*, *I mean it’s understaffed and it’s not just that one patient and you have to be there to do a fistula-dressing change for one-two hours a couple of times a day*, *so it just doesn’t work in terms of staff*. ***MD***: *And of course*, *it’s in the toughest stage when*, *before you get it to work*. *Sooner or later it works*, *it [the fistula] heals but of course it could take a month before you get there and just like you say*, *the daily fistula-dressing take like*, *two hours*. ***ET***: *And of course*, *it takes such a short time when the dressing change doesn’t work when there’s leakage*, *and perhaps you might not have time to stay there for two hours and instead you add more bandage*. *So*, *it [intestinal content] lies there and then everything is thoroughly destroyed*. *So*, *everything goes quite quickly when you destroy it [the skin]*.

[Interview 2]

The enterostomal therapy nurses in the hospitals often felt alone in working with the fistula dressing and that there was no one who could relieve them from their work. The nurses and nurse assistants in the hospital or at home contacted the enterostomal therapy nurse for help when the dressing did not work and the enterostomal therapy nurse was often asked for advice. The enterostomal therapy nurse felt a great responsibility to find an appropriate fistula dressing and failing to do so caused feelings of frustration.

The healthcare professionals working with home-based healthcare experienced challenges in making it as good as possible for the patient at home. Barriers that made care difficult were for example, lack of space or missing tools such as a suction device, which is a prerequisite for being able to do the fistula dressing. The need for multiple changes of the fistula appliance every day was difficult to meet when the travelling time to reach the patient was long. This problem was even more pronounced with leakage through the fistula appliance, which promoted an urgent visit. There was an understanding by the healthcare professionals working at the hospital that it was not an easy task to provide care at home.

***HRN***: *But then of course*, *I have also had the benefit of working in home healthcare in the evenings and at night and facing the complexity of what it means to care for them here at home*. *Because I remember from the earlier inpatient care*, *how complex it was to report on the patient and didn’t understand how they*, *how they manage to receive them here at home*? *Mm*, *when you can barely manage it on a medical ward*, *then you have to take care of it… with a long distance to the patient’s home*, *a long way from the hospital*.

[Interview 1]

#### Lacking disease-specific knowledge and skills

The more staff involved in the care, the greater the challenges faced that everyone has adequate knowledge and skills of the condition and situation of each specific patient. Due to the complexity and rareness of the condition knowledge and skills were often lacking, for example, in practical aspects such as flushing the fistula. This was regardless of working place and sometimes led to the patient not receiving the care needed. It became a heavy responsibility for the small group of knowledgeable healthcare professionals who often became the ones who had to provide most of the care and act as supervisor for colleagues with less knowledge. For the less experienced, it was important to have someone to discuss and learn from otherwise it could lead to longer periods of care. Unfortunately, the competence of experienced nurses and nurse assistants in hospital and home-based healthcare was not always available. The enterostomal therapy nurse expressed that they wanted to support the home healthcare professionals in how to properly dress the fistula. It was an advantage if home-based healthcare team could visit the patient while in hospital but this was not always possible. Another way to gain successive learning, was to work in pairs during the dressings, but this was very resource consuming and often not possible in reality.

***ET***: *Then of course*, *I can feel that it’s also part of my job as an enterostomal therapy nurse that you have to find a thing that anyone should be able to do*. *It mustn’t be too complicated*. *Because then it’s too time-consuming for the staff in the home who are supposed to help*, *so there’s a lot of stuff and… that has to work*.

[Interview 3]

As ECF is a rare condition, it was considered difficult to acquire the knowledge and skills needed about the disease and how to care for these patients. Regarding fistula dressing, participants expressed the need for more training and that they had to learn over time. When a written care plan was available, it facilitated care and saved time. Having knowledge of the condition and the specific care plan for the individual made it easier to communicate with and motivate the patient. Lack of knowledge could lead to non-compliance with the care plan and care was undertaken differently depending on which professional was working.

***HRN***: *It made it incredibly easy for me as a nurse that I know the illness trajectory*, *I´m able to communicate with my patient*. *Yes*, *but of course we are now in this phase and what you can do as a patient*, *is optimise not only the nutrition but also the activity*, *so the mobilisation and avoid infection again*. *That is*, *how you can encourage and motivate and get the patient to understand their own care and that process then takes time*. *But it took a while before because I didn’t learn any of that in any nursing education*, *we never got to talk about that*. *But it’s something that you have learnt from experience*.

[Interview 1]

### Caring requires an integrated approach

The complexity of these patients required an integrated approach. Both in the hospital and in home-based healthcare, it was considered necessary to work as an integrated team with different disciplines. That was not always easy and could lead to collaborative problems and challenges within the working group. Barriers were especially notable in collaboration between hospitals and home-based healthcare and the transfer of information between different care providers was also a challenge.

#### Challenging to work interprofessionally

Participants stated that collaboration between different professions and competences was necessary both in the hospital and in home-based healthcare.

***ET***: *The interprofessional collaboration is important because otherwise you’ll get nowhere*, *you can’t help the patient yourself but it requires you to collaborate with physicians and dieticians and everyone*.

[Interview 4]

Both in hospital and in the home-based healthcare there was a lack of specific team meetings for these patients. This led to fragmented care and the goal of care could be viewed differently by different team members. There was often a lack of a long-term planning leading to treatment delays. As patients with ECF are treated for a long time both in the hospital and at home, there were often different staff and actors involved in the care of the patient, which hindered the care process moving forward. A general experience was that it was easier to provide care if there were fewer staff involved but with adequate information about the patient’s situation and the goal of the care. However, working in a team was not always easy, especially when the patient was cared for at home.

Participants stated that it was important for the working group that they had the same starting point in the care of these patients; that ECF is a disease with no quick fix. A lack of common goal could lead to contradictions. For example, some surgeons described that there could be times when different specialties did not agree on the care. Participants also experienced that there sometimes was a lack of commitment to this patient group. It felt burdensome and unfair for those healthcare professionals with the most experience in caring for patients with ECF when they always needed to take on a greater responsibility, compared to others who were not as committed. Conflict in the group of nurses and assistant nurses arose when they treated the same patient differently. Some of the participants were more flexible because of patient preferences while others strictly followed the care plan.

***NRN 1***: *After all*, *we are in their home and it’s my opinion that if the patient wants the bandage to be a little more to the left*, *then we move it a little more to the left*. *We had*, *not conflict in the working group*, *but a bit more of difference in approach*. *Perhaps some thought that you should do it in exactly this way*, *instead of following the advice of the patient and then there would be conflict and the patient would just end up really*, *really annoyed… and I can understand that of course because the patient is the expert in her own fistula-dressing*. ***HRN 2***: *It’s her body*. ***HRN 1***: *There was a big conflict within the working group when we perhaps did a little of what the patient said in the fistula-dressing change and then some didn’t just because it is supposed to be done in a particular way*..

[Interview 5]

#### Facing barriers in collaboration between providers

Information transfer between healthcare professionals within the hospital and between the hospital and home-based healthcare was stated as a problem in the provision of integrated care. Similarly, the different medical record systems in the hospitals and in home-based healthcare further hindered collaboration between providers for example, in planning the discharge of a patient. The physicians and nurses in the hospitals often felt that everything was clear but the nurses in home-based healthcare services felt planning was complex and unclear. Although the hospital nurses spent a lot of time planning the discharge, their experience was that no matter how much they tried there was a high risk of readmission. For example, due to lack of an appropriate fistula dressing.

***MD***: *Those with a large volume of flow in the fistulas*, *then it becomes a problem as soon as they come from the hospital*, *as there is of course no one to monitor the flow and then they come in dehydrated and with electrolyte imbalances*. *It is difficult enough to manage outside the hospital*. *Unless it’s not such patients who are feeling well enough and can measure and monitor it*. ***RN***: *Of course*, *it’s often those who come in due to problems with the bandaging as well and there has been leakage or similar and they’re completely skinned and… it becomes complicated that way*. *And then they’ll be hospitalised for a while*. ***ET***: *That’s right and then you have to kind of start all over again*. *The skin has to heal so that it [the fistula appliance] can stay in place and so on*. *It’s a bit like that…*

[Interview 3]

Contact with hospitals was perceived as difficult for home healthcare professionals and there was a lack of feedback from the hospital on long-term planning for the patient. The home-based healthcare professionals wanted to have a contact person in the hospital and it was their experience that it was difficult to reach the attending physician, especially while the patient was waiting for reconstructive surgery. A lack of continuity among physicians delayed decisions regarding care. From the perspective of the home-based healthcare professionals, it required a lot of work to provide care for a patient with ECF and they emphasised that collaboration between different actors in the community was required to meet the significant needs of the patients.

***HRN***: *Yes it works*, *but it requires a lot of planning*, *there is often a lot of material that needs to be distributed to the patient´s home*. *There’s a lot of stuff around these patients*. *It also requires a lot from the family*, *a huge amount of acceptance from them*. *Because wherever it is*, *if the fistula is in the abdomen there might not be so much of an odour as there is if it is coming from the rectum and things like that*. *Or the bladder*, *so there’s a lot of things… a lot of acceptance from them*, *from families*. *And we solve it*, *but it’s quite resource demanding*. *There are a lot of visits*, *a lot of visits per day perhaps*, *the bandages don’t last*. ***HAN***: *Collaboration with the municipal homecare service*. ***HRN***: *We need to have collaboration with many actors otherwise we wouldn’t be able to manage it*..

[Interview 4]

### Building long lasting relationships is fundamental to the caring process

Caring for a patient with ECF meant building a relationship with the patient and the families over a long time, sometimes up to several months or years. Several participants described the relationship in a positive way, where the patient became a member of the team. But sometimes it was also challenging to maintain a professional approach. The healthcare professionals needed to balance all impressions and feelings that arose around management of the ECF. They felt insecure, inadequate, and uncomfortable when providing care. Even if it was sometimes challenging, participants emphasised the importance of involving the patient and their close family in the care in order to maintain a good relationship. Participants also acknowledged the patient’s suffering and placed great importance on commitment and availability, which was fundamental to the caring process.

#### Hiding your own feelings

Participants described that they sometimes found it challenging to hide their own feelings from the patient when the ECF smelled bad and looked unpleasant. Frustration when the fistula dressing did not hold without leaking was also difficult to hide from the patient. There were often several hours of work with the appliance, and it was frustrating not being able to adequately help the patient. They had to accept that the appliance worked one day but the next day did not work at all. Participants described feeling an inner stress in these situations and it became challenging to maintain professionalism.

***AN***: *And just not show when the bandage hasn’t held*, *to not show frustration*: *“Oh no*, *now I have to do this all over again”*. *These fistulae also often smell really bad*.

[Interview 1]

The healthcare professionals were involved in all aspects of the patient’s whole life. Angry, sad, and frustrated patients and family members had a negative effect on the healthcare professionals, which led to feelings of inadequacy. It was also stressful to be caught in the middle of a family that were negatively affected by the disease. In such situations, it was hard to remain professional and it challenged the relationship with the patients and their families. Knowing that the patient’s condition could last for several months, meant it was sometimes hard to keep the mood of patients up.

***MD***: *I really feel that it is just this patience*, *and that patience always runs out somewhere during the journey*. *Much connected to the fact that there is frustration among the staff*, *why is nothing happening*, *nothing is getting any better and then a frustration*, *perhaps with the patient and family that it’s really difficult to have someone who in principle*, *is basically living on a ward for six months or three months*.

[Interview 2]

Over time, patients often became highly involved in their care and often became “experts” on their own disease. Some participants expressed that they felt they were being questioned by the patient and that the patient did not trust them. This led to feelings of insecurity and was experienced by healthcare professionals both in the hospitals and in home-based healthcare. There was a need for a debrief of the working group around these patients but this was rarely carried out. Participants describe it as being important to talk openly in the working group about uncertainties in the care of a patient with ECF.

***ET***: *It’s the case with most of these patients*, *they are really… well*, *they are watching and they see exactly what you are doing and then are like*: *"No but you can’t put the bandage like that*, *because she put it over there and it worked well*, *so why are you doing it like that*?*"*. *And then you’re really interrogated and questioned many times during the fistula-dressing*.

Interview 2]

Challenging situations with patients in pain were experienced by all participants. At home, most patients took care of their own pain treatment while in hospital it was the nurse’s job to provide extra analgesics when needed. Participants experienced that many of these patients require large amounts of analgesics and sometimes it was difficult to manage this problem.

#### Involving the patient and their close family member

The healthcare professionals became involved in all aspects of the patient’s entire life, which also included their families. Involving the patient and family in the care was considered important for building long-lasting relationships. Participants described how they gained a close personal relationship with the patients and their families. All participants were willing to go the extra mile for these patients and often spent a little extra time with them.

***ET***: *You get much closer than you get to the other patients who don’t spend as long time on the ward*. *I´m following this woman to the ostomy clinic and then*, *well then they share a bit with you*, *yes of their life*. *And then you become a bit closer*. *I think that this relationship between care recipient and caregiver becomes bit of a grey zone*, *they become a bit like a friend*. *You know the patient by their first name*, *it becomes a different kind of relationship*, *I don’t know how you would describe it but…* ***RN***: *Of course*, *yes it could be something for example*, *like they ask me on Monday morning*: *"Oh so what did you get up to in the weekend*?*"…* ***ET***: *But she explained about her wedding*, *holiday plans*, *I knew where the patient had their holiday home*, *that the grandchildren would be visiting*, *things like that*. ***HRN***: *Yes*, *you get to know a lot…* ***RN***: *And perhaps they also find out a bit more about us because we have such a long relationship…* ***ET***: *Yes*, *exactly*, *it’s a different kind of relationship*..

[Interview 4]

Participants report that building trust between caregiver and patient and their families was considered important for establishing a long-lasting relationship. The patients became experts on their disease, and it was important to listen to the patient. The patient could often guide the healthcare professionals for example, in fistula dressing. It was also experienced as positive to see when it worked out well for patients to be at home after a long hospital stay.

It was difficult to have a general care plan as each patient with an ECF had a unique need for care. For example, the plan for fistula dressing needed to be individualised. There was often a lack of patient and family involvement in care planning while in hospital. In contrast, the healthcare professionals working in the patient´s home felt that they planned the care together with the patients. They let the patient decide a lot about their care and they saw the patient as an expert on their own body and illness. Individual adaptations meant that even if there were prescriptions for how care should be provided, the healthcare professionals often had to be creative and change the care plan over time.

***AN***: *I feel that you have to be a little inventive*, *you try to come up with different solutions*. *Something that the enterostomal therapy nurse has prescribed or that must be in a certain way but when I get there*, *I might find that it doesn’t work*. *That you… yes*, *well that you can’t just stick with that fistula-dressing but that you have to try to find something else all the time*, *something that is better*.

[Interview 2]

Participants described becoming very involved with their patients. An important work task was encouraging and motivating the patient to have the strength to fight despite the negative feelings involved with the condition. To meet patients’ needs when they for example had requests for when the fluids should be administered or when healthcare professionals should make home visits, was considered important.

The participants stated that patients felt secure when they were cared for in hospital and it was a big step for them to leave the hospital and be cared for at home. Given the great need for care even after discharge, the healthcare professionals felt that it would be of value for the patient to have a contact person at the hospital. Such a contact nurse was rarely found.

#### Acknowledging patient´s suffering

Participants had a great understanding of the patient’s situation and experienced that suffering was constantly present. They often found the patients feeling down and how they seemed to have lost control over their own lives. The participants also experienced that patients often expressed a feeling of insecurity and ashamed about leakage of stool. There was a great understanding of the wide range of things that affected patients negatively for example, pain and the risk of opiate addiction.

***RN***: *Naturally*, *there’s a lot of fear behind all of these things*, *there’s the fear that it will hurt when you are mobilised*, *because it’s difficult to relieve the patient’s pain and find a long-acting medicine that works well*. *Then of course*, *it’s also difficult with the actual fistula-dressing*, *eating and drinking can be a challenge*, *just daily activities*, *talking to people—there’s a lot of shame and guilt that can be behind this as well*. *Of course it’s hard to be bedridden with a fistula with leakage of stool*. *So there is really a lot of factors that come into play in the full picture*..

[Interview 2]

Participants expressed a keenness to make things as good as possible for the patients and to maintain as much quality of life as possible. It was considered important to be able to give patients hope and facilitate things for them as much as possible in everyday life.

***ET***: *What you want is for it to be as bearable as possible for them*. *Because it’s a pretty nasty situation*, *irrespective of where the fistula is or whether it’s big or small*, *the fact is that they have it and naturally the quality of life*, *of course it has an enormous impact on quality of life*. *So I probably feel that in my job I am trying to do as much as possible to make it a bearable life situation*.

[Interview 3]

## Discussion

To our knowledge, this is the first study to date that explores the experiences of healthcare professionals in providing care for patients with ECF in hospital and in home care. The main findings are that caring for patients with ECF is complex and consumes time and resources, requires an integrated approach and that building long lasting relationships is fundamental to the caring process. Based on our findings, the clinical experience in the research group and the literature, we have suggested different strategies for healthcare professionals for the facilitation of care of patients with ECF–a patient group that has complex medical and nursing needs, as described in Table 4.

**Table 4:** Supportive strategies for healthcare professionals to facilitate the care of patients living with enterocutaneous fistula.

Findings	Level ^a^	Strategies ^b^
Struggling with practical issues	Organisation	Ensure that all equipment for the home is ordered at the time of discharge for example, a suction device Schedule sufficient time for each patient
Lack of disease-specific knowledge and skills	Organisation	Provide a photographic fistula dressing plan and a written care plan that follows the patient at discharge Implement peer learning during fistula dressings and care of the patient to facilitate successive learning Embed evidence-based guidelines into daily clinical practice
Challenging to work interprofessionally	Team	Conduct team meetings regularly and discuss challenges and disagreements within the team to avoid fragmented care and treatment delays Provide written long-term planning so that all team members are working towards the same goal
Facing barriers in collaboration between providers	Organisation	Strengthen the process around discharge and arrange for staff working in the home-based healthcare district to visit the patient in hospital Provide a contact nurse at the hospital for staff working in the home-based healthcare district Define roles and distribute tasks among team members Organise care through continuity of healthcare professional as much as possible
Hiding your own feelings	Organisation	Provide a supportive environment for healthcare professionals working with patients with enterocutaneous fistula Arrange time for professional reflection in groups
Involving the patient and their close family member	Team	Emphasise central role of patient in managing their health Use effective self-management support strategies that include assessment, goal setting, action planning, problem-solving and follow-up Perform person-centred care and provide a home-based care plan in collaboration with the patient and family Provide a contact nurse at the hospital, as well as in home care for the patient and family
Acknowledging the patient´s suffering	Team	Continue to give patients hope and facilitate everyday living as much as possible Consider involvement of a psychologist or counsellor in the team when needed Pay attention to the patient’s pain while simultaneously being aware of a possible opiate addiction

^a^ Level = team and/or organisation;

^b^ the strategies should not be considered as evidence based clinical guidelines.

We found that struggling with practical issues such as finding an appropriate fistula dressing for the patient or not have the right equipment to carry out the care was challenging and complicated the care process. Furthermore, lack of space at home, a long distance to the home of the patient and working with different medical record systems all had a negative effect on the healthcare professionals. These practical problems lead to feelings of inadequacy and that the work of the care providers became unsatisfactory and stressed. Reducing negative feelings among team members is important because it can otherwise increase the risk of developing ethical stress when there is a feeling that the patient is not being given safe and proper care [15,16]. Our findings emphasise the importance of an equal working environment for the healthcare professionals, irrespective of where the patient is being cared for. Healthcare organisations should consider the range of hindrances encountered by healthcare professionals in their work and how it impacts stress levels and the ability to do the best for their patients. Furthermore, our findings also suggest that knowledge of the patient’s condition and the skills to carry out nursing procedures were considered necessary to facilitate care—the lack of such could lead to team members feeling incompetent and patients not receiving the care they needed. This reasoning is supported by a study by Kvarnström et al. [17] which stated that it could be harder to collaborate effectively and deal with the patient’s situation if the knowledge about the patient differ within the team. Kvarnström et al. [17] also found that interaction in the team can facilitate interprofessional learning and that learning is strengthened when difficulties are managed in an open joint discussion. In the present study, participants reported a lack of team meetings, which might lead to a suboptimal organisation of care. As the care of patients with ECF requires a multi-professional collaboration both within the team, as well as between healthcare professionals working in the hospital and home-based healthcare [1,2,5,9], regular meetings are of the greatest importance. However, bringing professionals together in teams will not automatically lead to collaboration and professionals must trust each other before a collaborative process can be established [17]. This is important to have in mind as one barrier to collaboration in our study was in fact a lack of trust between healthcare professionals and their different prerequisites for providing healthcare in hospital and in the patient’s home. Having regular team meetings could clarify perceived challenges within the team, which thereby enhances interprofessional collaboration [17].

Furthermore, we found that all participants stated that caring for patients with ECF requires an integrated approach but that this was hard to establish in reality. The World Health Organisation (WHO) [18] states that integrated healthcare should be centred on the needs of the individuals and their families and delivered by a coordinated multidisciplinary team of providers, working across settings and levels of care. A recent systematic literature review [19] found that models of integrated care may enhance patient satisfaction, increase perceived quality of care, and enable access to services. However, shortage of staff or lack of continuity with a number of different care providers being involved in the care, is a well-known problem in healthcare services that has emerged as an obstacle to practicing integrated care [20]. One suggestion to facilitate to an integrated care process in working with this patient population could be to strengthen the process around discharge, which was experienced as challenging in the present study. Slater [21] points out that discharge planning should commence as early as possible as patients need to be prepared emotionally and going home can be daunting. It is important that all equipment and products required for management of the ECF are available when the patient returns home so healthcare professionals can provide the planned care. In accordance with the results of the present study, Slater [21] discusses encouraging the community nursing team to visit patients while they are still a patient in hospital. This practice allows the community nursing team to meet the patient and observe a fistula dressing, enabling them to develop awareness of how they can support the patient at home. Establishing a contact nurse after discharge for both healthcare professionals and patients is also of utmost importance.

Finally, we found that it was fundamental for the healthcare professionals to build long-lasting relationships with the patient and the family, as this is a group of patients that will suffer the ECF for a long time. All patients and their families had unique needs and therefore it could not be assumed that everyone with an ECF should be cared for in the same way. One way to facilitate the individual needs is through person-centred care in which the individual is seen as a decision-making, valuable and equal partner in the planning, execution and follow-up on care [22,23]. To perform person-centred care, it is necessary to have knowledge about the person behind the patient, their lifeworld, their experience of illness and the consequences of symptoms and treatment. The partnership and trust between healthcare professionals and patients and their families is fundamental [22,23]. The healthcare professionals in the present study described a great commitment to these patients but that it sometimes was challenging to establish a relationship over time, as the condition can lead to frustrations for not only the patient but also the family and the healthcare professionals. Pain management was something that could be frustrating for the nurses. For example, many of the patients required large amounts of analgesics and when they were at home, they took care of their own pain treatment while in hospital it was the nurse’s job to provide extra analgesics when needed. This work task was not always easy since it sometimes could be difficult to assess whether the patient had “true” pain or whether there was a risk of opiate addiction. Horner et al. [24] found similar results in their study when they interviewed nurses about their attitudes and training needs when caring for patients with comorbid opioid use disorder. The authors discussed the importance of working as a team and having the same goal with the patient’s treatment. It helps if pain management is made clear in the individual care plan, especially in the transition between hospital and homebased healthcare [24]. Overall, the healthcare professionals in our study acknowledged patients’ suffering and were aware of the negative impact that the ECF had on the daily life of both the patient and their family and tried to meet the needs and requests of the patient. This is in agreement with one of our previous qualitative longitudinal studies showing that patients with ECF and their family members faced an unpredictable and restricted life with social isolation and a lot of physical and psychological symptoms [7].

### Strengths and limitations

The Lincoln and Guba [25] framework of trustworthiness in qualitative research was applied in this study. To ensure the dependability of the study, the sampling procedure and analysis followed the method step-by-step [25]. Credibility of the interview guide was assured through the conduct of a pilot-interview [13]. The dynamics and interpersonal relationships in the focus groups, as well as different experiences, knowledge and education could have influenced what each participant felt could be talked about in front of others. A mixed gender focus group could be a limitation, as men tend to speak more frequently and with more authority than women and the moderator had an important role in guiding the interview [11]. To increase the trustworthiness of findings, analytic triangulation was undertaken [25,26]. Quotations were used to illuminate the findings and to verify categorisation [13,25]. The findings may not be generalised and applicable to all healthcare professionals caring for these patients as the experience can differ for geographical reasons and if the experience is based solely on the care of one patient. On the other hand, the findings may likely be transferred to Sweden and other countries with a similar context. From the beginning, the plan was to have mixed focus groups with participants from both hospitals and home-based healthcare. Due to the pandemic, this mix could not be conducted in all focus groups, which is why the study was supplemented with a fifth focus group containing only nurses from a home-based healthcare district.
